# Scleroderma with crescentic glomerulonephritis: a case report

**DOI:** 10.1186/1752-1947-2-151

**Published:** 2008-05-13

**Authors:** Arunachalam Ramaswami, Thiraviam Kandaswamy, Tholappan Rajendran, Kizhake Pisharam Jeyakrishnan, Hla Aung, Mohammaed Iqbal, Chakko K Jacob, Haji Shaukat Zinna, Gazala Kafeel

**Affiliations:** 1 Department of Nephrology, RIPAS Hospital, Bander Seri Begawan, Brunei Darussalam; 2 Department of Pathology, RIPAS Hospital, Bander Seri Begawan, Brunei Darussalam

## Abstract

**Introduction:**

Systemic sclerosis or scleroderma is an autoimmune rheumatic disease characterized by organ-based fibrosis. Renal involvement in scleroderma occurs mainly in the form of scleroderma renal crisis, affecting 5 to 10% of patients. It remains one of the most important and immediately life-threatening complications of scleroderma, but the prognosis improves considerably after treatment with angiotensin-converting enzyme inhibitors. Other renal pathologies can occur in scleroderma. These include scleroderma overlap syndromes with associated features of lupus nephritis, myeloperoxidase anti-neutrophil cytoplasmic antibodies (ANCA) or proteinase 3 ANCA-associated glomerulonephritis, or crescentic glomerulonephritis. These alternative pathologies should be suspected in any individual patient with a differing clinical picture and the patient should be appropriately investigated. Crescentic glomerulonephritis occurs very rarely in scleroderma. This report describes a patient with scleroderma and crescentic glomerulonephritis.

**Case presentation:**

A 52-year-old woman with a known history of scleroderma and hypertension on angiotensin-converting enzyme inhibitors was referred to the nephrologist because of a rapid decline in renal function. Kidney biopsy was performed which revealed immune complex type crescentic glomrulonephritis. Cytoplasmic-staining ANCA was negative. Despite immunosuppressive treatment the patient rapidly went into end-stage renal failure and is still on hemodialysis.

**Conclusion:**

Scleroderma is a complex disease, and the best characterized renal involvement in scleroderma is scleroderma renal crisis. However, other renal pathologies can occur in scleroderma. These alternative pathologies should be suspected in any patient with a differing clinical picture and the patient should be appropriately investigated, as the clinical course and treatment are different from the more common scleroderma renal crisis.

## Introduction

Scleroderma (systemic sclerosis) is a chronic systemic disease that targets the skin, lungs, heart, gastrointestinal tract, kidneys and musculoskeletal system. The disorder is characterized by three features: tissue fibrosis, small blood vessel vasculopathy and a specific autoimmune response associated with autoantibodies. Scleroderma is classified into two major subsets, diffuse and limited cutaneous sclerodermas, that are distinguished by the extent of skin thickening. Diffuse scleroderma is characterized by widespread skin thickening involving distal and proximal body regions; rapid onset (within 1 year) of skin and other features following appearance of Raynaud's phenomenon; significant visceral involvement; high scores on disability and organ damage indices secondary to extensive fibrosis of tissues associated with antinuclear antibodies; and the absence of anticentromere antibody. Limited scleroderma shows limited skin thickening, slow progression of disease and late visceral involvement, with unique features of isolated pulmonary hypertension and digital amputations associated with anticentromere antibody. Overlap syndromes have diffuse or limited scleroderma features plus features typical of one or more other connective tissue or autoimmune diseases. Mixed connective tissue disease shows features of scleroderma, systemic lupus erythematosus polymyositis, rheumatoid arthritis and the presence of anti-U1 sn-RNP antibodies.

Approximately 10% of patients with scleroderma have a renal crisis that mimics malignant hypertension, with rapidly progressive renal failure secondary to microvascular disease, vasospasm and tissue ischemia. Microangiopathic hemolytic anemia and thrombocytopenia can accompany scleroderma renal crisis. Studies demonstrate high levels of serum renin levels associated with vasospasm and intrinsic renal vessel disease. A renal crisis is associated with the use of corticosteroids or can be precipitated by conditions compromising renal blood flow (dehydration). Any hypertension (> 140/90 mmHg) in a scleroderma patient should be carefully evaluated because a renal crisis is potentially reversible with appropriate management with angiotensin converting enzyme (ACE) inhibitors. Patients presenting with serum creatinine above 270 μmol/l have a poor prognosis. Some patients who progress to renal failure and dialysis can recover renal function after months of dialysis therapy.

Variable changes may be seen in the glomeruli. In some cases thickening of glomerular capillary walls with a double contour appearance on silver or periodic acid-Schiff staining may be seen. Fibrinoid necrosis may also be seen. Crescents are very rare and those that are seen are invariably small. Interlobular arteries show intimal thickening which is mucinous or finely fibrous. The thickening results in a considerable reduction of the lumen.

Crescentic glomerulonephritis (GN) represents a severe form of glomerular disease that is characterized by disruption of the glomerular basement membrane, leading to cellular proliferation in the Bowman's space and is often accompanied by fibrinoid necrosis. Crescentic GN is classified into three major types. Anti-glomerular basement membrane (anti-GBM) disease is characterized by circulating anti-GBM antibodies and linear deposition of antibodies along the glomerular basement membrane. This constitutes around 10% of cases. Pauci-immune (anti-neutrophil cytoplasmic antibodies (ANCA)-associated GN) is characterized by scanty glomerular deposits of immunoglobulin and circulating ANCA, and comprises about 60% of cases. Immune complex-mediated GN is a heterogeneous group of diseases usually associated with obvious granular deposits of immunoglobulins, in which crescent formation complicates an identifiable form of nephritis, usually proliferative in type. This constitutes around 30% of cases. The causes of immune complex-type crescentic GN include infection (including hepatitis C virus (HCV) associated cryoglobulinemia), systemic immune complex diseases (especially systemic lupus erythematosus) and underlying pre-existing primary GN.

In a study of crescentic GN [1] the underlying etiology was as follows: ANCA-associated vasculitis 37%; systemic lupus erythematosus 23%; IgA nephropathy 12%; mesangiocapillary GN 6%; focal segmental GN 6%; anti-GBM disease 6%; postinfectious GN 3%; membranous GN 2%; focal segmental glomerulosclerosis 2%; Henoch Schonlein purpura 1%; others 3%.

The mainstay of therapy has remained immunosuppression with combinations including steroids, cyclophosphamide and azathioprine with or without methylprednisolone, and plasma exchange [2]. In lupus nephritis with crescents, cyclophosphamide is useful, and mycophenolate mofetil is a useful alternative. In anti-GBM disease and ANCA-associated nephritis with severe renal failure plasma exchange is used. Plasma exchange removes pathogenic autoantibodies but the process also has an effect on inflammatory mediators and possibly cell-mediated immune function.

Myeloperoxide ANCA-associated normotensive crescentic GN in scleroderma has been reported from Japan. We report a case of scleroderma with crescentic GN.

## Case presentation

The patient is a 52-year-old Asian woman who presented to the rheumatology department of our hospital in August 2003 with complaints of tightness of the skin over the face and hands. She also had positive Raynaud's phenomenon. She was diagnosed with scleroderma. She did not have any difficulty swallowing. Fundus examination did not reveal any evidence of accelerated hypertension.

Her investigations revealed ++ urine protein, ++ white blood cell (WBC), 10 to 20 RBC/hpf (red blood cells per high power field) and her 24-hour urine protein was 1.17 g. Her blood investigation revealed a WBC count of 7.5 × 10^3 ^hemoglobin of 9.9 gm/dl, a platelet count of 378 × 10^9 ^and erythrocyte sedimentation rate of 78 mm in the first hour. Total bilirubin was 11 μmol/l, alanine transaminase was 10 units/l; she was HBSAg (hepatitis B surface antigen) negative and anti-HCV antibody negative.

She was also antinuclear antibody (ANA) positive (1 in 640). Her blood urea was 7.9 mmol/l, S-Creatinine 135 μmol/l, sodium 141, and potassium 4.8. Anti-RO, -LA, -Sm, and -RNP antibodies were negative. Anti-ds DNA antibodies were negative. Lupus erythematosus cells were negative. Anti-Scl70 antibodies were positive. Cytoplasmic-staining (C)-ANCA was negative. Perinuclear-staining (P)-ANCA was reported as 'unable to determine because of positive ANA' (P-ANCA testing by immunoassay would have clarified this inconclusive result but was unavailable to us). Rheumatoid factor was less than 8IU/ml. (reference range (RR) < 30IU/ml). Serum C3 was 0.76 g/l (RR 0.88 to 2.01 g/l). Serum C4 was 0.32 g/l (RR 0.16 to 0.58 g/l).

X-ray of the hands showed acro-osteolysis of the terminal phalange of thumb and index finger. High-resolution computed tomography of the lungs revealed prominent bilateral sub-pleural honeycombing suggestive of usual interstitial pneumonia. A Doppler study of the renal arteries did not reveal any evidence of renal artery stenosis.

**Figure 1:**
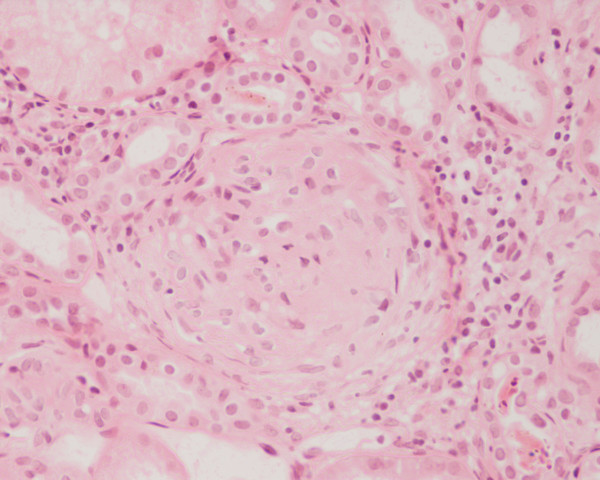
**Photomicrograph of the renal biopsy showing prominent fibrocellular crescent formation and moderate mesangial proliferation in a glomerulus**. Hematoxylin and eosin stain.

**Figure 2:**
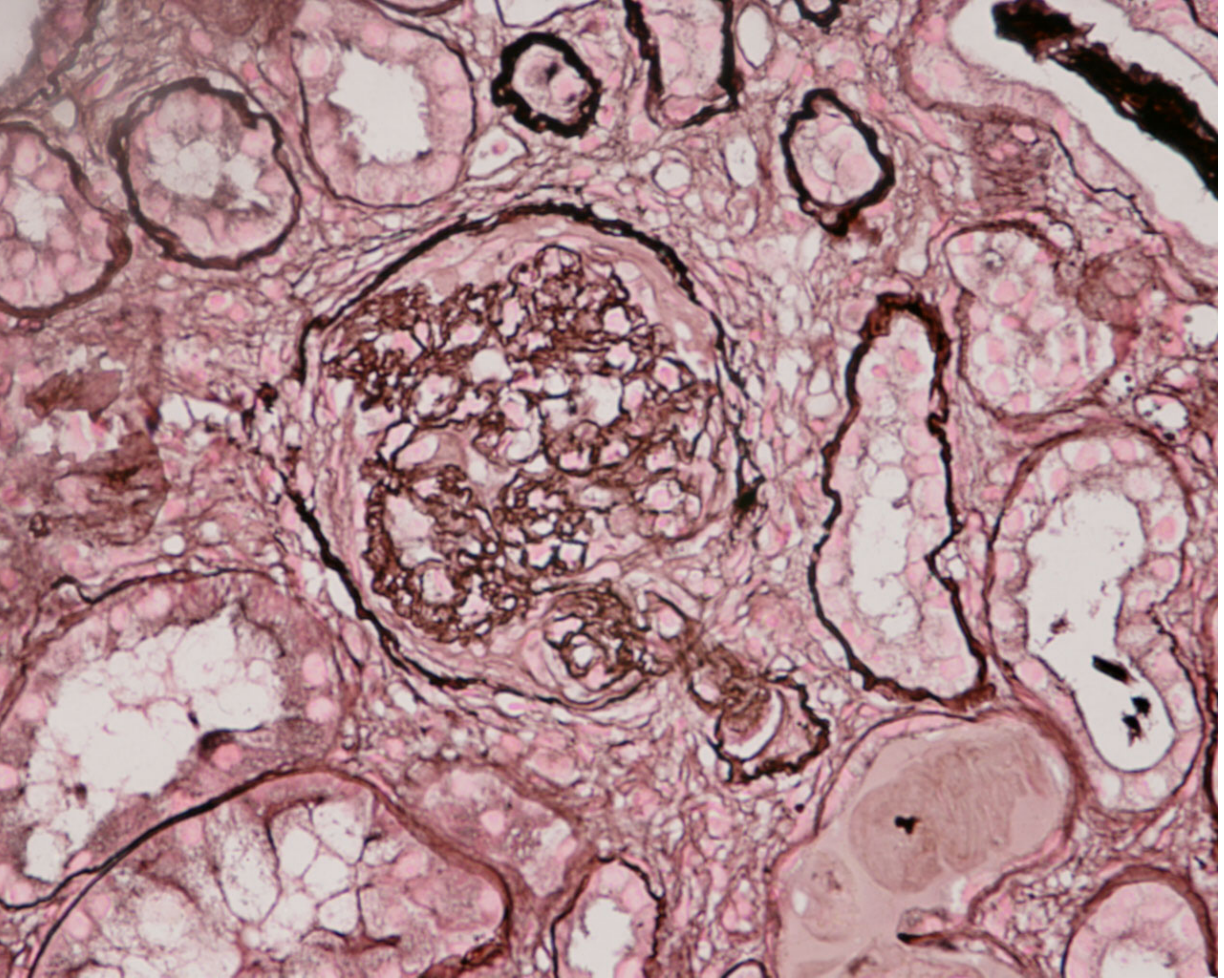
**Photomicrograph of renal biopsy showing crescent formation and tuft narrowing**. Periodic acid silver methanamine stain.

**Figure 3:**
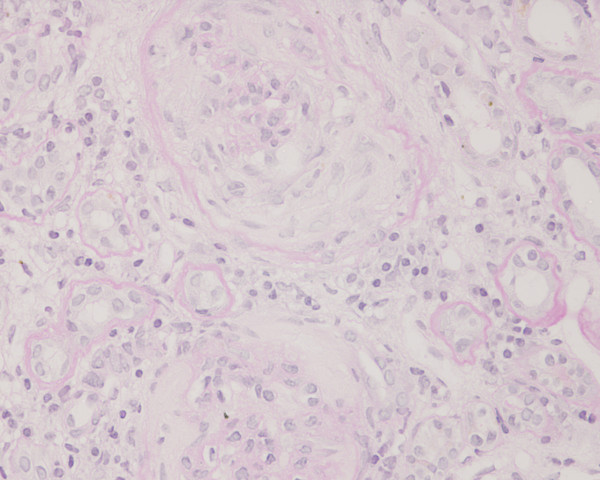
**Photomicrograph of the renal biopsy showing prominent fibrocellular crescent formation**. Chronic inflammatory cellular infiltration and fibrosis is also evident in the interstitium. Periodic acid-Schiff stain.

The patient was diagnosed with diffuse scleroderma, treated symptomatically and given ACE inhibitors for hypertension. In June 2004 she was referred to a nephrologist. At that time her investigations were as follows: blood urea 22.7 mmol/l, serum sodium 137, serum potassium 6.8, and serum creatinine of 555 μmol/l. Ultrasonogram examination of the abdomen revealed echogenic kidneys of normal size. In view of the relatively rapid decline in renal function and normal size kidneys a renal biopsy was performed (Figures 1, 2 and 3).

A kidney biopsy was performed which showed 26 glomeruli. Most of the glomeruli (95%) showed prominent fibrocellular and fibrous crescent formation and the lesions were global. Capillary wall basement membrane thickening and duplication with tram track appearance and moderate mesangial proliferation were evident. The glomerular hypercellularity was mainly mesangial with occasional polymorphs with no necrotizing lesion. Five glomeruli were completely sclerosed. The extraglomerular renal vessels exhibited moderate thickening of the vessel wall, particularly of its medial layer. Chronic inflammatory cellular infiltration and fibrosis of interstitial tissue were also seen. Immunoperoxide staining showed marked positivity with ++ IgG, + IgM, ++ Focal C3 and negative IgA. The staining pattern was granular in the mesangium and peripheral capillary loops. The overall impression was of crescentic GN with granular immune deposits.

Therapy with methylprednisolone was not effective and the patient went into end-stage renal failure and was initiated on hemodialysis on 14 July 2004. She is still on maintenance hemodialysis three times per week.

## Discussion

Scleroderma is a connective tissue disorder with multi-organ involvement. Numerous symptoms in scleroderma such as Raynaud's phenomenon, digital tip necrosis and angina are related to ischemic changes attributable to intimal thickening of medium and small arterial vessels in the absence of histopathologic changes of vasculitis. Renal involvement in scleroderma is due to diminished blood flow in the afferent arterioles and is called scleroderma renal crisis. This includes acute deterioration of renal function, hypertension, and hyperreninemia and absent nephritic urinary sediment. It usually responds to ACE inhibitors.

Histopathologically there is concentric edematous intimal thickening of interlobular arteries and fibrinoid arteriolar necrosis causing elevation of plasma renin level with onset or aggravation of hypertension and rapid deterioration of renal function as a result of ischemic glomerulopathy [3]. There are well recognized co-occurrences of other acute renal pathologies in scleroderma. This is especially seen in scleroderma overlap syndromes with lupus nephritis. In such cases there may be histological clues such as development or rise in titer of anti-ds DNA antibodies [1]. Crescentic GN is an uncommon cause of renal failure in scleroderma but it has been observed in patients treated with D-penicillamine. This agent is not widely used but has been associated with antibody generation, including ANCA.

Reports from Japan describe patients with long-standing scleroderma and myeloperoxidase ANCA positive normotensive crescentic GN [4]. The clinical features of patients with crescentic GN are different from those with scleroderma renal crisis. The treatment requirements of these two conditions also differ [5]. Those with crescentic GN require immunosuppressive drugs such as methylprednisolone and cyclophosphamide and they do not respond to ACE inhibitors [6].

There is a recently described condition with features of scleroderma called nephrogenic systemic fibrosis (nephrogenic fibrosing dermopathy) that develops in patients with advanced kidney disease, apparently in association with gadolinium exposure. Even though it is described mainly in patients with end-stage renal disease (ESRD) on dialysis, it can occur in individuals with a glomerular filtration rate of <30 ml/minute. Due to this, restricted use of gadolinium-enhanced MRI has been recommended. Our patient does not have a history of exposure to gadolinium.

Our patient presented with renal involvement within one year of diagnosis of scleroderma. She did not respond to ACE inhibitors because she did not have classical scleroderma renal crisis. She did not respond to immunosuppressive treatment with methylprednisolone. She rapidly went into ESRD and became dialysis-dependent. Although recovery from dialysis-dependent renal failure is possible in crescentic nephritis, this has not occurred in this patient. This case is presented for the rarity of crescentic GN in scleroderma and the different clinical course from scleroderma renal crisis.

## Conclusion

Scleroderma is a complex disease with multisystem involvement. The best characterized renal involvement in scleroderma is acute or sub-acute renal hypertensive crisis (scleroderma renal crisis), but other renal pathologies can occur. These include scleroderma overlap syndromes with features of lupus nephritis, myeloperoxidase ANCA, proteinase 3 ANCA-associated GN or crescentic GN. These alternative pathologies should be suspected in any individual patient with a differing clinical picture and the patient should be appropriately investigated because the clinical course and treatment are different from the usual scleroderma renal crisis.

## Abbreviations

ACE: angiotensin converting enzyme; ANCA: anti-neutrophil cytoplasmic antibodies; anti-GBM: anti-glomerular basement membrane; ESRD: end-stage renal disease; GN: glomerulonephritis; HCV: hepatitis C virus; RR: reference range; WBC: white blood cell.

## Competing interests

The authors declare that they have no competing interests.

## Authors' contributions

AR, TK, TR, KPJ, HA, MI, CKJ, HSZ wrote or contributed to the writing of the manuscript. GK processed the kidney biopsy specimen and wrote the biopsy report.

## Consent

Written informed consent was obtained from the patient for publication of this case report and accompanying images. A copy of the written consent is available for review by the Editor-in-Chief of this journal.
